# Insulin resistance in non-diabetic patients of chronic Hepatitis C

**DOI:** 10.12669/pjms.291.2888

**Published:** 2013

**Authors:** Zareen Kiran, Bader Faiyaz Zuberi, Daniah Anis, Rashid Qadeer, Khalid Hassan, Salahuddin Afsar

**Affiliations:** 1 Dr. Zareen Kiran, MBBS, Department of Medicine, Dow University of Health Sciences, Karachi, Pakistan.; 2 Dr. Bader Faiyaz Zuberi, FCPS, Department of Medicine, Dow University of Health Sciences, Karachi, Pakistan.; 3 Dr. Daniah Anis, MBBS, Department of Medicine, Dow University of Health Sciences, Karachi, Pakistan.; 4 Dr. Rashid Qadeer, FCPS, Department of Medicine, Dow University of Health Sciences, Karachi, Pakistan.; 5 Dr. Khalid Hassan, FCPS, Department of Medicine, Dow University of Health Sciences, Karachi, Pakistan.; 6 Prof. Salahuddin Afsar, FRCP, Department of Medicine, Dow University of Health Sciences, Karachi, Pakistan.

**Keywords:** Insulin Resistance, Chronic Hepatitis C Infection, Diabetes Mellitus, HOMA-IR

## Abstract

***Objective:*** To determine insulin resistance in non-diabetic chronic hepatitis C patients using Homeostatic Model Assessment of Insulin Resistance (HOMA-IR).

***Methodology:*** Patients having anti-HCV positive were included in this study. Patients with diabetes mellitus, thyroid disease, hyperlipidemias, hypercortisolism and infective diseases other than hepatitis C were excluded. Age, weight, height and absence of diabetes were documented. Fasting blood glucose and fasting insulin levels were done. Body mass index and insulin resistance was calculated using the formulas. Patients having insulin resistance using formula HOMA-IR>2.5 were labeled as insulin resistant. Data was analyzed using SPSS-18.

***Results:*** One hundred and fifty five patients according to sample size estimation were enrolled, in whom HOMA-IR was calculated, the mean value was found to be 2.47 ±1.30. A total of 79 (51%) of patients had HOMA-IR more than 2.5 showing insulin resistance.

***Conclusion: ***In a third world country like Pakistan, where there is a high prevalence of hepatitis C infection, the consequences of the disease are also very common. Insulin resistance was found in 51% of patients with chronic hepatitis C.

## Introduction

 Insulin resistance is one of the gravest metabolic disturbances of human body, growing rapidly all over the world. It is an important marker of metabolic syndrome in general, and is an independent risk factor for its cardiovascular complications. It is also very well known to be associated with chronic hepatitis C infection.^1^ Hepatitis C is the most common cause of chronic hepatitis worldwide, which causes liver damage over a course of time. The chronic low grade inflammation in patients with chronic hepatitis C leads to persistent immune cell activation and inflammatory cytokine release causing metabolic disruption in adipose tissues.^2^

 The hepatic steatosis triggered in patients with hepatitis C is similarly the consequence of these metabolic derangements, that is insulin resistance, and is responsible for accelerated fibrosis and low response rate in hepatitis C treatment-naïve patients.^3^^,^^4^ One of the recent trials have shown improvement in insulin resistance on treating hepatitis C.^5^ Moreover, insulin resistance has also been found to be a major determinant of the outcome of patients who are treated for hepatitis C.^6^ Studies have been conducted regarding the importance of insulin resistance in management strategy of hepatitis C^7^^,^^8^ and its complications like hepatocellular carcinoma.^9^ Therefore, to effectively manage patients with chronic hepatitis C, it is important to address insulin resistance as part of their management protocol.

 Homeostatic Model Assessment of Insulin Resistance (HOMA-IR) is a simple clinical tool which can be easily used in epidemiological studies to determine insulin resistance. Our region also has high prevalence of chronic hepatitis C and its clinical complications along with fore-coming treatment and management difficulties, which signifies the need to determine the insulin resistance as an important parameter in management strategies.

## Methodology

 A total of one hundred and fifty five patients, who were either attending medical unit II OPD or were admitted in ward were enrolled in the study after taking informed consent. Inclusion criteria included patients who were anti-HCV positive by EIA. Patients with the following conditions were excluded from the study: ischemic heart disease, diabetes mellitus, thyroid disease, hyperlipidemias, hypercortisolism and infective diseases other than Hepatitis C. Data was collected for age, gender, weight, height, medical history including absence of diabetes. BMI was calculated and patients were allocated to normal, under and overweight categories accordingly. Fasting blood samples were obtained to determine the fasting blood glucose and fasting insulin levels (by radioimmunoassay) from a single laboratory. Insulin resistance was determined for all patients using HOMA-IR, as under:^10^

$\mathrm{HOMA}-\mathrm{IR}=\frac{\mathrm{Fasting Glucose x Fasting Insulin}}{405}$

 Patients with HOMA-IR > 2.5 were labeled as insulin resistant and those with HOMA-IR < 2.5 as noninsulin resistant group. Data analysis was done using SPSS version 18.0. Descriptive statistics were calculated for all continuous variables. Mean ± Standard Deviation was calculated for age, weight, height, BMI, Fasting blood glucose levels and fasting Insulin levels. Frequencies & percentages for categorical variables were calculated. The effect of independent variables upon the main outcome variable, that is, presence of insulin resistance based on HOMA-IR was calculated by Binary Logistic Regression. The Odds Ratio along with 95% confidence intervals was calculated.

**Table-I:** Univariate analysis for different variables.

*Variable*	*Insulin resistant (%)*	*Odds Ratio*	*95% CI*
*Age*			
25-40 yrs 41-55 yrs >55 yrs	41 49 100	0.12 0.52 1.00	0.02, 0.89 0.15, 1.06 --
*Gender*			
Male Female	51 52	1.01 1.00	0.52, 1.92 --
*BMI*			
Normal Overweight	50 66.7	0.50 1.00	0.21, 1.16 --
*FBS*			
Normal Impaired	50 66.7	0.78 1.00	0.34, 1.36 --

## Results

 A total of 155 patients with diagnosed HCV infection were enrolled in the study. The age ranged from a minimum of 18 years and a maximum of 60 years. The mean age was 40.6 years ±10.7 years. Majority of the patients were in the age ranges of 30-35 years (37%) and 50-55 years (20%). Of all 155 patients, 87 (56.1%) were males while 68 (43.9%) patients were females. Mean height was found to be 1.66 meters ±0.08 meters while mean weight came out to be 63.15 ±14 Kg. Body Mass Index was calculated as weight (in Kg) per height (in meters) squared. Mean BMI was calculated to be 23.82 ±6.31 kg/m^2^. Of all 155 patients, 93 (60%) had BMI more than 25 kg/m^2^, thus obesity was found to be more frequent among the patients. Mean fasting glucose level in 155 patients was found to be 98.37 mg/dL with standard deviation ±19.45 mg/dl. A total of 60% patients had normal fasting glucose level, 30% of patients had impaired fasting glucose level. Whereas a total of 10% patients had fasting glucose level more than 126mg/dl or more.

 Mean fasting insulin levels in all patients were found to10.60 ±6.11 uU/ml. Of all 155 patients in whom HOMA-IR was calculated, the mean value was found to be 2.47 ±1.30. A total of 79 (51%) of patients had HOMA-IR more than 2.5 showing insulin resistance. When comparison was made with the independent variables with the presence of insulin resistance among the patients, following results were found. According to gender distribution, equal numbers of males & females were found to have insulin resistance. A total of 44 males & 64 females had insulin resistance calculated on the basis of HOMA-IR. According to age distribution, in age group 25-40 years of age 40% patients suffered from insulin resistance, while in age group 40-55 years 50% of patients suffered from insulin resistance. All patients aged 55 years and above had insulin resistance. When BMI was compared with the presence of insulin resistance a total of 66.66% patients who were overweight were found to have insulin resistance. Table-I shows the Odds Ratios for different variables after the univariate analysis.

## Discussion

 Insulin resistance is a growing pandemic all over the world. Hepatitis C virus is now found to be one of the causal factors of insulin resistance. On the other hand insulin resistance in patients with chronic hepatitis C (CHC) is responsible for development of type 2 diabetes later.^11^ Insulin resistance (IR) is also an independent risk factor associated with the development of hepatocellular carcinoma in hepatitis C patients.^12^ It has been reported to be high even in non-diabetic, non- cirrhotic CHC as compared to normal controls (62.3% vs 16.0%).^13^

In patients with hepatitis C, viral infection has already started the process of steatosis, which is secondary to IR. This leads to metabolic disturbance in the body and is a contributing factor to hepatitis C patient morbidity and mortality.^8^ It is necessary therefore to have an assessment of insulin resistance in such patients to manage them effectively. Moreover, the treatment of chronic hepatitis C patients may also be affected if their metabolic profile is simultaneously addressed.^14^ Insulin resistance and diabetes are common complications of all chronic liver diseases. However, several epidemiological, clinical and experimental data show that HCV plays a direct role in perturbing glucose metabolism, leading to both insulin resistance and diabetes.

 Most cross-sectional studies comparing the prevalence of diabetes in patients with chronic hepatitis C with that of a comparator group have shown that patients infected with HCV present with diabetes more often than patients with chronic liver diseases of other etiologies, even at a pre-cirrhotic stage.^15^ This observation was confirmed by a vast general population-based survey and by several longitudinal studies.^16^ Most of the risk affects patients with other cofactors of diabetes, suggesting that HCV infection may significantly increase the rate of developing glucose metabolism alterations in predisposed individuals. The association between HCV infection and glucose abnormalities holds true also when looking at the occurrence of pre-diabetes conditions, such as insulin resistance. Curing HCV results in the amelioration of insulin resistance and decreased incidence of diabetes after the end of therapy.^17^

 In this study the results found were comparable with the results of international studies. There was a very high frequency of insulin resistance (51%) was found in patients with chronic hepatitis C infection. Multiple factors were found to be associated with the presence of insulin resistance. Age greater than 55 years and BMI more than 25 kg/m^2^ were positively associated with insulin resistance. This is in accordance with international findings.^18^^-^^20^ Gender difference, on the other hand was not found to be associated with insulin resistance.

## Conclusions

 In a third world country like Pakistan, where there is a high prevalence of hepatitis C infection, the consequences of the disease are also very common. Insulin resistance was found in 51% of patients with chronic hepatitis C.
